# Effect of Various Beverages on Adhesion of Repaired CAD/CAM Restorative Materials

**DOI:** 10.3390/jfb14070380

**Published:** 2023-07-20

**Authors:** Elif Yiğit, Hamiyet Güngör Erdoğan, Tan Fırat Eyüboğlu, Mutlu Özcan

**Affiliations:** 1 Department of Prosthodontics, Faculty of Dentistry, University of Karabük, Karabük 78050, Türkiye; 2 Department of Prosthodontics, Faculty of Dentistry, Lokman Hekim University, Ankara 06510, Türkiye; gngr.hamiyet@gmail.com; 3 Department of Endodontics, Faculty of Dentistry, Istanbul Medipol University, Istanbul 34083, Türkiye; tfeyuboglu@yahoo.com; 4 Clinic of Masticatory Disorders and Dental Biomaterials, Center of Dental Medicine, University of Zurich, CH-8032 Zurich, Switzerland

**Keywords:** adhesion, composite resin, dental materials, polymer infiltrated ceramic network, resin matrix ceramic, resin nanoceramic, shear bond strength

## Abstract

(1) Background: The purpose of this study was to determine the effect of commonly consumed beverages on the bond strength of three different computer-aided design-computer-aided manufacturing (CAD/CAM) resin–ceramic hybrid materials repaired with resin-based composite (RBC) materials. (2) Materials and Methods: Rectangular prism specimens (N = 138) measuring 6 mm × 5 mm × 2 mm were obtained from GC Cerasmart (GC), Lava Ultimate (LU), and Vita Enamic (VE) blocks. These blocks were polished and then subjected to thermal cycling (10,000 cycles, 5 °C to 55 °C). After the surface treatment was applied, the average surface roughness value was measured. All the surfaces were repaired with RBC. Thermal cycling was performed for the second time. Each group was then distributed into three subgroups according to the beverage used: tea (t), cola (c), and distilled water (0) (*n* = 15). The specimens were stored in these solutions for 28 days and then subjected to the shear bond strength (SBS) test. Statistical analysis was performed using a two-way ANOVA test with Bonferroni adjustment. (3) Results: The surface roughness of the materials presented no significant difference after different surface treatments (*p* > 0.05). No significant difference was observed among the materials (*p* > 0.05). Tea and cola presented similar SBS values (*p* > 0.05). Both were significantly lower than distilled water (*p* < 0.001, *p* < 0.001, respectively). (4) Conclusions: Consumption of beverages reduces the bond strength in surfaces repaired with RBC to CAD/CAM resin–ceramic hybrid materials. (5) Clinical Significance: Repairing damaged resin matrix dental restorations with RBC is advantageous in terms of time and cost by achieving adequate bond strengths. Frequently consumed beverages reduce the bond strength of repaired CAD/CAM resin–ceramic hybrid materials.

## 1. Introduction

In recent years, computer-aided design/computer-aided manufacturing (CAD/CAM) techniques have gained significant popularity in dentistry. This is mainly due to advances in adhesive techniques and the introduction of new materials. CAD/CAM systems offer numerous advantages, including improved standardization of the restoration manufacturing process and reduced production costs [1]. CAD/CAM systems allow restorations to be fabricated in a single visit to the dentist. The materials used for CAD/CAM restorations are primarily divided into ceramics and composites. While ceramics are the preferred material for indirect restorations in clinical practice, they exhibit low fracture toughness and high brittleness [2].

Technological developments in dentistry, especially in ceramic materials, have made it possible to produce all-ceramic materials. Its qualitative properties, such as excellent esthetic appearance, natural tooth color, color stability, biocompatibility, chemical inertness, high flexural strength, fracture toughness, low thermal conductivity, and low wear resistance, make it superior to metal-supported ceramics [3]. However, the high hardness of ceramics can cause excessive wear on the antagonist tooth [4,5]. Ceramic restorations can have high failure rates due to their fragile structure [6]. In order to overcome these disadvantages, searching for the development of dental esthetic materials and polymer-based resin composites has been explored. However, traditional dental composites have polymerization shrinkage and poor mechanical properties. The need to develop a new material that combines the favorable properties of ceramics and resin composites is esthetic, durable, and does not cause wear on the antagonist tooth led to the development of CAD/CAM resin–ceramic hybrid materials [7,8]. CAD/CAM resin–ceramic hybrid materials cause less enamel wear on the antagonist tooth and offer more advantages than glass ceramics. GC Cerasmart (GC Dental Products, Kasugai, Japan), Lava Ultimate (3M ESPE, Seefeld, St. Paul, MN, USA), and Vita Enamic (Vita Zahnfabrik, Bad Sackingen, Germany) are recently introduced resin matrix ceramics. Lava Ultimate contains bisphenol A glycidyl methacrylate (Bis-GMA), bisphenol A diglycidyl methacrylate ethoxylated (Bis-EMA), urethane dimethacrylate (UDMA), triethylene glycol dimethacrylate (TEGDMA) with high heat content zirconia with nanoceramic structure placed in a polymerized resin matrix and silica filler particles. Vita Enamic consists of an alumina, silica, potassium oxide, sodium oxide, boron trioxide, calcium oxide, zirconium dioxide, and titanium dioxide feldspar matrix infiltrated with UDMA and TEGDMA containing polymers. GC Cerasmart combines the favorable properties of high-strength ceramics containing silica and barium glass with an resin-based composite (RBC) containing 2,2-bis (4 methacryloxy-polyethoxyphenyl) propane (Bis-MEPP), UDMA, and dimethacrylate (DMA) [9,10,11]. Despite all the advances in CAD/CAM materials, inadequate occlusal fit, internal stress, parafunction, and porosity during fabrication can cause fractures in the restoration [12]. Fractures are common in CAD/CAM restorations, regardless of whether they are made of ceramics or composites. When faced with such failures, clinicians have the choice of replacing the failed restoration or opting for a complete repair. Total replacement is not always the best option in many clinical situations because it can potentially damage healthy tooth tissue and is more time-consuming. Repairing the failed restoration has the advantage of minimizing time and reducing the risk of trauma to surrounding tissues. Repairing failed indirect restorations rather than replacing them is often the preferred approach. However, achieving a durable and reliable bond between the failed restoration and the RBC can be challenging. Restoration repair involves preparing the surface of the failed restoration and completing the missing portion with an RBC. The clinical success of the repair is highly dependent on the bond strength between the failed restoration and the composite resin [2].

Repairing ceramics with RBC is a tissue-friendly method that can be applied quickly to reduce treatment costs [13]. Prior to repairing ceramics with RBCs, surface preparation is required to ensure proper bonding [14]. Mechanical and chemical surface treatments have been proposed to provide adequate adhesion between the ceramic surface and the restorative repair material. Roughing with a diamond bur, sandblasting, acid etching, and laser can be cited as examples of mechanical surface treatments, while silane application can be cited as an example of chemical surface treatment [15,16].

Composite-based dental restorative materials are continuously exposed to various damaging factors in the oral cavity, resulting in potential changes in their fundamental properties. These factors can be classified as mechanical, chemical, and thermal. Chemical factors can be further classified as external (such as acids present in the air, acidic foods and beverages, or chlorinated water in swimming pools) and internal (including gastric acids from frequent vomiting). The presence of these acids can cause erosive damage to both the natural hard tissues of the teeth and the RBC materials used in dental restorations [17]. Hanging et al. demonstrated that the occurrence of erosion is influenced by the pH of a fluid, specifically in the range of two to three. In addition, even a small decrease in the pH value can lead to an escalation in the erosive potential of the fluids [18].

Beverages are known to have adverse effects on the restorations, such as discoloration and loss of bond strength. Previous studies have investigated the discoloration effect of various beverages and solutions on dental restorative materials [19,20,21,22]. In addition, many studies have investigated the mechanical, physical, and esthetic properties of CAD/CAM resin–ceramic hybrid materials [19,23,24,25,26].

However, to the best of our knowledge, there are no studies in the literature that investigated the effect of beverages on bond strength. This study aims to fill this gap and presents the experimental results obtained by applying commonly consumed beverages to the specimen to observe the effect on bond strength. The hypothesis tested was that different beverages would not significantly decrease the shear bond strength of various repaired CAD/CAM resin–ceramic hybrid dental restorations with RBC.

## 2. Material and Methods

The materials used in this study are listed in Table 1. Three different CAD/CAM resin–ceramic hybrid blocks, GC Cerasmart (GC), Lava Ultimate (LU), and Vita Enamic (VE), were used in this study. A total of 138 specimens (6 mm × 5 mm × 2 mm) were cut by using a high-precision low-speed water-cooled cutting device (Micracut 151, Metkon, Bursa, Türkiye). Each specimen was embedded in blocks of acrylic resin (Kemdent, Swindon, UK) and then ground and polished in cold running water using 600- and 1000-grit silicon carbide abrasive paper. After polishing, the specimens were cleaned using an ultrasonic cleaner (Everest Ultrasonic, Ankara, Türkiye). Prior to the repair process, the specimens were subjected to 10,000 thermal cycles at temperatures of 5 ± 2 °C and 55 ± 2 °C (Esetron, Ankara, Türkiye). The specimens were left in the tanks for 30 s and then transferred to the other tank within 5 s.

The surface of each specimen was abraded and roughened with a green banded diamond fissure bur for four seconds with horizontal and vertical movements to ensure standardization. After every five specimens, the bur was replaced with a new one [27,28]. The average roughness value (Ra) was measured using a contact profilometer (Perthometer M2, Mahr, Göttingen, Germany). The roughness value was determined by calculating the average of the measurements taken on three different parts of the specimens. The profilometer was recalibrated after every ten measurements. After measuring the surface roughness, the specimens from each group that were closest to the average roughness value were selected for scanning electron microscope analysis. The surface morphological characteristics of the samples were analyzed by scanning electron microscopy (SEM; JSM-5600 LV, JEOL, Tokyo, Japan). Each sample was coated with gold–palladium, and the SEM images were taken at 100× and 1000× magnification.

**Figure 1:**
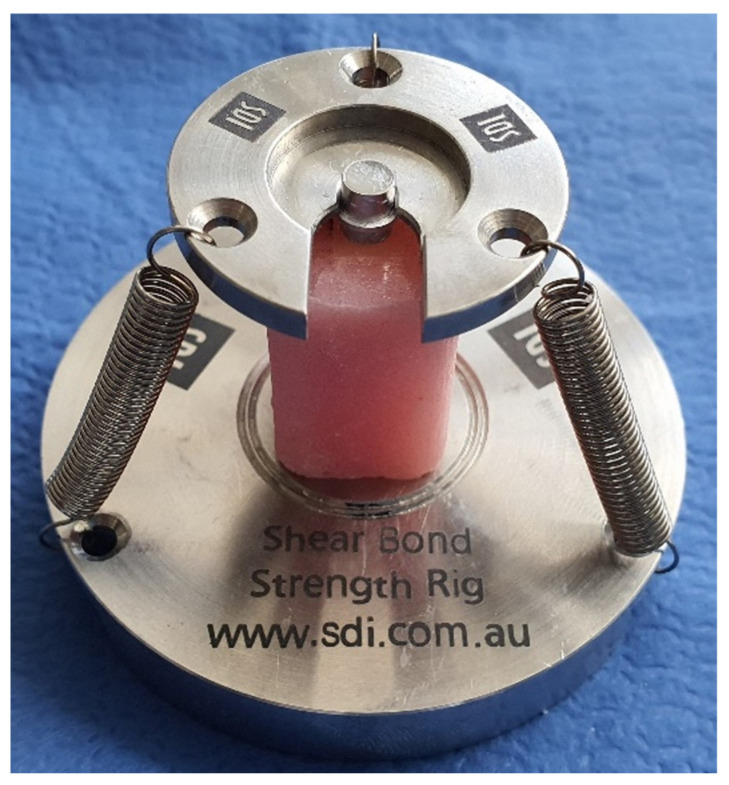
Image of a specimen in the SDI SBS Rig test apparatus.

Prior to placement of the restorative material, silane (G-Multi Primer, GC Corporation, Tokyo, Japan) was applied on the specimens with a brush for 1 min and dried with oil-free air. A thin layer of universal bonding agent (G-Premio Bond, GC Corporation, Tokyo, Japan) was applied to the entire surface for 10 s. Excess material was removed with an airflow for 5 s, and then the adhesive was polymerized with a light-emitting diode (LED) curing device light irradiance at 1470 mW/cm [2] (Elipar, 3M ESPE, Seefeld, Germany) for 10 s. After the application of the adhesive system, each acrylic specimen was placed in a shear bond rig (SDI Shear Bond Strength Rig, SDI Limited, Bayswater) to provide a common bonding area (Figure 1). A hollow cylindrical stainless-steel mold with an inner diameter of 3.5 mm and a height of 5 mm was placed over the bond area of each specimen, aligning the center of the mold with the center of the specimen surface. The mold was then gradually filled with ultrafine hybrid RBC (GC Essentia Universal GC, Tokyo, Japan) using the incremental technique in two layers of 2 mm each, and each layer was light cured with an LED curing device (Elipar S10 3M ESPE, Seefeld, Germany) for 20 s. Silane, adhesive system, and RBC were applied according to the manufacturer’s instructions. After polymerization, the cylindrical mold was removed, and further polymerization was performed. The specimens were stored in distilled water at 37 °C for 24 h. After the repair process, the specimens were thermocycled for 10,000 cycles.

After thermocycling, each group was further divided into three subgroups (*n* = 15) to be stored in distilled water (control) (0), tea (Lipton Yellow Label, Istanbul, Türkiye) (t), and cola (Coca-Cola, Istanbul, Türkiye) (c). The samples were kept in these solutions for 28 days, and the solutions were renewed daily.

The shear bond strength (SBS) test was performed using a knife edge chisel (Lloyd LF Plus; Leicester, England) at a crosshead speed of 1 mm/min until failure. The following formula was used to calculate the maximum stress: Stress (MPa) = Load (N)/Area (mm^2^). After the application of SBS, the failed surfaces of the specimens were examined under a stereomicroscope (NZ.1902-P, Euromex, Arnhem, The Netherlands) at 10× magnification, and the failure modes were classified as adhesive, cohesive (cohesive in ceramic), and mixed fracture.

Statistical analysis of the collected data was performed using IBM SPSS Statistics 29 (SPSS Inc., IBM Corp., Armonk, NY, USA). The Shapiro–Wilk test was used to analyze the homogeneity of the data for both roughness bond strength analyses. Two-way analysis of variance (two-way ANOVA) was used to determine whether there was a difference between more than two groups in the bond strength analyses. When performing ANOVA, Levene’s test for variance homogeneity is inspected first. Bonferroni adjustment was used to test for differences between the groups in the variables. For the surface roughness analysis, one-way ANOVA was used to determine whether there was a difference between more than two groups, with Bonferroni adjustment for pairwise comparisons. Chi-squared test was used to evaluate the difference in failure mode types between materials and beverages.

## 3. Results

The statistical power of the between-subject effects was found to be 99%, with a large effect size in the pairwise comparisons of material and beverage (0.196), which provides sufficient statistical power with the present sample size.

**Figure 2:**
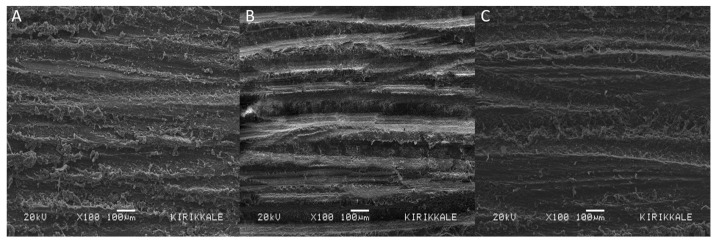
SEM images of the bur-ground specimens. (**A**) GC, (**B**) LU, (**C**) VE. The composite phase was observed as dark gray, while the ceramic phase was observed as light gray regions.

The average surface roughness values among the materials did not show a statistically significant difference (*p* > 0.05) (Table 2). During the SEM examination, rough, deep grooves, small hills, depressions, and protrusions were observed on the material surfaces of all three different CAD/CAM resin–ceramic hybrid materials (Figure 2).

The highest SBS value was observed in the following order: VE-0 > LU-0 > GC-c > GC-0 > LU-t > GC-t > VE-c > LU-c > VE-t (Table 3). The SBS test results were homogeneously distributed in both materials and beverages. No significant difference was observed between materials (*p* > 0.05), while there was a significant difference between beverages (*p* < 0.001). The difference between tea and control (*p* < 0.001) and between cola and control (*p* < 0.001) was significant. However, there was no significant difference between tea and cola (*p* > 0.05). The highest SBS value was observed in VE-0, followed by LU-0, GC-c, GC-0, LU-t, GC-t, VE-c, LU-c, and VE-t (Table 3). According to the beverage-wise comparisons, LU-c had significantly lower SBS values than LU-0 (*p* < 0.001). VE-0 had significantly higher SBS values compared to VE-c (*p* < 0.001) and VE-t (*p* < 0.001). (Table 3).

Adhesive failure was more common in the tea and cola groups than in the control group. However, mixed failure type was mainly observed in the control group (Table 4). In this study, for GC Cerasmart, adhesive failure types were seen as most common in GC-0, and cohesive and mixed failure was more common in GC-t and GC-c. For Lava Ultimate, LU-0 experienced mostly cohesive and mixed failures, and LU-t experienced mostly mixed failures. LU-c experienced all failure types equally. VE-0, VE-t, and VE-c all experienced mostly mixed failure (Table 4). Statistical analyses showed no significant difference between materials and within the beverages (*p* < 0.05).

## 4. Discussion

In this in vitro study, the quantitative and qualitative effects of the common beverages on the bond strength between three different CAD/CAM resin–ceramic hybrid materials and RBC were evaluated. It was found that the beverages studied significantly affected the bond strength values when an RBC was bonded to three different CAD/CAM resin–ceramic hybrid materials. According to the results of this study, the null hypothesis was rejected.

As a result of developments in esthetic dentistry, CAD/CAM resin–ceramic hybrid materials have been developed that combine the favorable properties of ceramics and composites. Compared with traditional ceramics, the modulus of elasticity is closer to dentin and easier to mill and arrange than glass matrix or polycrystalline ceramics, and repairs and modifications can be easily performed with composite resin. Albero et al. [29]. reported that CAD/CAM resin–ceramic hybrid materials have flexural strength closer to natural tooth tissue than conventional ceramics. Chavali et al. [30] investigated the tensile and fracture strength of CAD/CAM resin–ceramic hybrid materials, lithium disilicate, and zirconia samples and found that resin matrix ceramics showed less failure than the other groups. Another advantage of using CAD/CAM resin–ceramic hybrid materials in esthetic dentistry is that they can be finished and polished with polishing kits without the need for a firing process to obtain a final restoration. In addition, they have a more flexible structure than glass ceramics and can absorb the chewing forces more evenly in posterior region restorations [9,10,11]. CAD/CAM resin–ceramic hybrid materials are preferred in the fabrication of dental restorations because of their mechanical and esthetic properties. If a restoration fractures for any reason, it is less costly and time-consuming to repair the restoration intraorally than to replace it. Dental restorations are constantly exposed to food and beverages due to daily eating habits. Although many studies have investigated the bond strength between RBC and CAD/CAM resin–ceramic hybrid materials [19,23,24,25,26], no study has investigated the effect of commonly consumed beverages on the bond strength between the two materials. This study investigated the influence of commonly consumed beverages in everyday life on the bond strength of CAD/CAM resin–ceramic hybrid specimens repaired with RBC.

In the present study, bur roughening was selected as a surface treatment method not only because it is easy to apply but also because it can provide sufficient surface roughness and adequate bond strength without the need to remove the restoration when repairing in the mouth. There are many examples in the literature of the use of bur roughening as a surface treatment prior to repair [19,23,26,31,32,33,34]. Previous studies have reported that the application of surface treatments results in improved adhesion of CAD/CAM resin–ceramic hybrid restorations [15,16,23,35,36,37]. The ideal surface treatment varies depending on the composition of the restorative material, and there is no clear consensus in the literature regarding the perfect surface treatment [38,39]. While the application of hydrofluoric acid after silanization seems to be the most appropriate surface treatment method for polymer-infiltrated nanoceramics, roughening with a diamond bur or air abrasion with Al_2_O_3_ is the most accepted/common surface roughening method for resin nanoceramics [40]. The application of hydrofluoric acid creates enough space for the ceramic surface and releases hydroxyl groups that provide bonding [41]. However, the use of hydrofluoric acid carries a risk due to its toxic effects. It can cause serious damage to soft tissues, skin, and lungs [39]. On the other hand, sandblasting with aluminum oxide is another commonly used surface roughening method. It produces a rough, irregular, and clean surface and increases the surface energy [24,42]. Sandblasting can be performed both outside and inside the mouth by using special sandblasting devices. However, there is a risk of inhalation of small-sized aluminum oxide particles when using an intraoral blasting device for sandblasting [43]. When the present study is evaluated in terms of surface roughness, the average surface roughness values of GC Cerasmart, Lava Ultimate, and Vita Enamic materials are, respectively, 2357 µm, 2370 µm, and 2353 µm. Although the highest surface roughness value belongs to the GC Cerasmart material, there is no significant difference between the groups in terms of surface roughness. Previous studies using bur grinding for surface treatment reported rough, deep grooves; small hills; depressions; and protrusions on the material surfaces during SEM examination, and these surface irregularities increased the bond strength between the RBC and the ceramic surface [19,23]. In all cases, surface treatment should be performed prior to the repair process to achieve a stronger bond strength between the two materials.

In this study, MDP (10-methacryloxydecyl dihydrogen phosphate) ceramic primer, which contains a silane coupling agent, was applied after the surface roughening. Silane coupling agents positively affect the chemical bond by increasing wettability and reducing the contact angle [25,38]. It has been reported that the application of silane increases the bond strength between the ceramic surface and the RBC after mechanical surface roughening processes [26].

Many studies have investigated the color changes caused by commonly consumed beverages in dental restorative materials. However, there is a need for a study in the literature that examines the effect of these beverages on the bond strength of dental restorative materials. Tea and cola are two of the most commonly consumed beverages in Türkiye [44,45]. For this purpose, in the present study, the repaired specimens were kept in distilled water, tea, and cola solutions for 28 days. It has been reported that if a glass of beverage is consumed for 15 min, keeping it in solution for 28 days is equivalent to more than two years [19].

In the present study, the bond strength values varied between 14–19 MPa regardless of the beverage. It has been reported that the optimum bond strength value for the composite resin is 15-25 MPa, depending on the RBC and repair method [23,35]. In the present study, similar bond strength values were observed for each group, which are clinically acceptable according to the literature [2,23,42,46]. Güngör et al. [23] applied different surface treatments to GC, LU, and VE materials and evaluated the shear bond strength. In the grinding group, the bond strength values were found to vary between 15–21 MPa, and the average bond strength values were similar to the present study. Bayındır et al. [47]; investigated the shear bond strength of different adhesive systems and surface treatments on GC, LU, and VE materials and reported bond strength values in agreement with our study. According to the results of the present study, the materials showed similar bond strength values. Demirtag and Culhaoglu [42]; applied various surface treatments to CAD/CAM resin–ceramic hybrid materials and evaluated the bond strength. They reported similar bond strength values similar to the present study.

Based on the results of this study, it can be stated that intraoral repair of CAD/CAM resin–ceramic hybrid restorations would provide sufficient bond strength and prevent replacement of the restoration. Bond strength tests are commonly used in in vitro studies to test the bond between ceramic and restorative materials and to provide information on the clinical performance of these materials. Shear forces are one of the forces commonly encountered in the mouth and can cause dislocation of the restorative material. For this reason, the shear test has clinical significance and is considered one of the most widely used tests to evaluate the bond strength of restorative materials [2,48,49,50].

In this study, the beverages tested decreased the bond strength between ceramic surfaces and RBCs. It was observed that the bond strength decreased significantly in the tea and cola groups. This is attributed to the thermal difference created by the tea and the acidity caused by the low pH value of the cola. Szalewski et al. [17] reported that popular beverages affect the microhardness and flexural strength of composite resins. Colombo et al. [51] evaluated the change in surface microhardness of resin nanoceramic materials after exposure to cola, and they reported that cola affected the mechanical properties of resin nanoceramic materials and caused the loss of microhardness.

After the shear test, the fractured specimens were examined under a stereomicroscope to determine the relationship between the bond strength values of the CAD/CAM resin–ceramic hybrid materials and the failure types, and the failure types were determined. It has been reported in the literature that mixed and cohesive failures after shear testing have higher bond strengths than adhesive failures and that cohesive and mixed failures are more common in macro tests than in micro tests [42,52]. In accordance with the literature, most of the cohesive and mixed failure types were observed after shear testing in the present study, while adhesive failures were more common in the tea and cola groups compared to the control group; the mixed failure type was mainly observed in the control group. These results support that tea and cola decreased the bond strength between the CAD/CAM resin–ceramic hybrid material and RBC.

The bond strength between ceramic and restorative dental materials may be affected by the type, structure, dimensions of the restorative material, surface treatment, ceramic type, aging procedure, and bond strength test method [53,54]. This situation causes different bond strength values in different studies, making them difficult to compare with each other. The limitations of this study are the inability to fully mimic the oral environment in terms of heat and humidity and the inability to measure the volume loss caused by the surface treatment applied to the resin matrix ceramic surface. However, further studies are needed in which oral fluids, including saliva, should be tested at different time intervals to evaluate the extent of the decrease in SBS over time, along with the effect of surface treatments on fracture resistance and color change of CAD/CAM resin–ceramic hybrid restorations prior to clinical trials.

## 5. Conclusions

Based on the results of this study, the following conclusions were drawn:

1. Tea and cola significantly reduced the repair bond strength of RBC to CAD/CAM resin–ceramic hybrid materials.
2. Intraoral repair of CAD/CAM resin–ceramic hybrid restorations with RBC provides adequate bond strength regardless of the beverage or CAD/CAM resin–ceramic hybrid material used.

## Figures and Tables

**Table 1:** The composition of the materials used in this study.

Material	Batch Number	Type	Composition
GC Cerasmart (GC Dental Products, Tokyo, Japan)	2007221	Resin Nanoceramic	Resin nanoceramic (Bis-MEPP, UDMA, DMA) with 71 wt% silica and barium glass nanoparticles
Lava Ultimate (3M ESPE, St. Paul, MN, USA)	N880844	Resin Nanoceramic	Bis-GMA, UDMA, Bis-EMA, TEGDMA with 80% wt 20-nm silica and 4- to 11-nm zirconia nanoparticles, and zirconia/silica nanoclusters
Vita Enamic Vita Zahnfabrik (Bad Sackingen, Germany)	74760	Hybrid ceramic	UDMA, TEGDMA. Filler: Feldspar ceramic enriched with aluminum oxide, 86% by weight
G- Multi Primer (GC Dental Products, Tokyo, Japan)	2101151	Silane	Silane, MDP, ethanol
G-Premio Bond (GC Dental Products, Tokyo, Japan)	2011181	Universal adhesive	MDP, 4-MET, MEPS, methacrylate monomer, acetone, water, initiators, silica
GC Essentia Universal (GC Dental Products, Tokyo, Japan)	200915A	Ultrafine hybrid	BisEMA (10–25 wt%), TEGDMA (2.5–5 wt%), UDMA (1–2.5 wt%), BisGMA (1–2.5 wt%), Melamine/formaldehyde resin (0.5 wt%), Butylated hydroxytoluene (<0.2 wt%) Nanofiller 81 wt%

Bis-GMA: A-glycidyl methacrylate, Bis-EMA: bisphenol A diglycidyl methacrylate ethoxylated, UDMA: urethane dimethacrylate, TEGDMA: triethylene glycol dimethacrylate, Bis-MEPP: 2,2-bis (4 methacryloxy-polyethoxyphenyl) propane, DMA: dimethacrylate.

**Table 2:** Comparison of the average surface roughness.

Material	Average Surface Roughness Value (μm)				*p*
	Mean	SD	Min	Max	
Lava Ultimate	2.370	0.283	1.871	2.707	0.984
GC Cerasmart	2.357	0.262	1.920	2.805	
Vita Enamic	2.353	0.291	1.862	2.762	
Total	2.360	0.273	1.862	2.805	

*p* < 0.05.

**Table 3:** Shear bond strength values (MPa) and standard deviation (SD) according to restorative beverages.

	Tea Mean ± SD (MPa)	Cola Mean ± SD (MPa)	Control Mean ± SD (MPa)
Lava Ultimate	16.935 ± 1.768 ^1^	15.141 ± 1.640 ^1^	18.353 ± 1.866 ^1,2^
GC Cerasmart	16.833 ± 1.728 ^1^	17.501 ± 1.449 ^2^	17.143 ± 2.204 ^1^
Vita Enamic	14.926 ± 2.283 ^2^	15.341 ± 1.352 ^1^	19.592 ± 3.359 ^2^

Different numbers indicate significant differences in columns, *p* < 0.05.

**Table 4:** Failure modes according to groups.

Groups	Failure Mode		
	Adhesive *n*	Cohesive *n*	Mixed *n*
GC-0	5	7	3
GC–t	2	5	8
GC–c	2	4	9
LU-0	1	7	7
LU-t	4	5	6
LU-c	5	5	5
VE-0	1	6	8
VE-t	3	3	9
VE-c	4	3	8

0: control, t: tea, c: cola; *n*: number of specimens.

## Data Availability

The data presented in this study are available on request from the corresponding author. The data are not publicly available due to ethical restirictions.
